# A Single Herpes Simplex Virus 1 Genome Reactivates from Individual Cells

**DOI:** 10.1128/spectrum.01144-22

**Published:** 2022-07-11

**Authors:** Dor Rafael, Enosh Tomer, Oren Kobiler

**Affiliations:** a Department of Clinical Microbiology and Immunology, Sackler School of Medicine, Tel Aviv University, Tel Aviv, Israel; University of Wisconsin-Madison

**Keywords:** fluorescence, genetic recombination, herpes simplex virus, herpesviruses, quiescent infection

## Abstract

Latent infection is a characteristic feature of herpesviruses’ life cycle. Herpes simplex virus 1 is a common human pathogen that establishes lifelong latency in peripheral neurons. Symptomatic or asymptomatic periodic reactivations from the latent state allow the virus to replicate and spread among individuals. The latent viral genomes are found as several quiescent episomes inside the infected nuclei; however, it is not clear if and how many latent genomes are able to reactivate together. To address this question, we developed a quiescent infection assay, which provides a quantitative analysis of the number of genomes reactivating per cell, in cultured immortalized fibroblasts. We found that, almost always, only one viral genome reactivates per cell. We showed that different timing of entry to quiescence did not result in a significant change in the probability of reactivating. Reactivation from this quiescent state allowed only limited intergenomic recombination between two viral strains compared to lytic infection. Following coinfection with a mutant that is unable to reactivate, only coreactivation with a reactivation-proficient recombinant can provide the opportunity for the mutant to reactivate. We speculate that each individual quiescent viral genome has a low and stochastic chance to reactivate in each cell, an assumption that can explain the limited number of genomes reactivating per cell.

**IMPORTANCE** Herpesviruses are highly prevalent and cause significant morbidity in the human and animal populations. Most individuals who are infected with herpes simplex virus (HSV-1), a common human pathogen, will become lifelong carriers of the virus, as HSV-1 establishes latent (quiescent) infections in the host cells. Reactivation from the latent state leads to many of the viral symptoms and to the spread of the virus among individuals. While many triggers for reactivation were identified, how many genomes reactivate from an individual cell and how are these genomes selected remain understudied. Here, we identify that, in most cases, only one genome per cell reactivates. Mutated HSV-1 genomes require coinfection with another strain to allow coreactivation. Our findings suggest that the decision to reactivate is determined for each quiescent genome separately and support the notion that reactivation preferences occur at the single-genome level.

## INTRODUCTION

Herpes simplex virus 1 (HSV-1) is a large, double-stranded DNA virus. It is estimated that 60% to 95% of the world’s population is seropositive to HSV-1 (1). HSV-1 is a neurotropic virus that establishes lifelong latency within sensory neurons (2). HSV-1 enters the human body usually through mucosal tissue, replicates productively within mucosal epithelial cells, and enters sensory neurons through nerve termini (3). At the sensory neurons, a latent infection can be established, which provides a viral reservoir for periodic reactivation. The virus can cause symptoms such as cold sores or genital lesions. Severe cases that are less frequent include keratitis, which may lead to corneal blindness and life-threatening encephalitis. Acyclovir (ACV), which is the most common antiviral drug in use today, helps in reducing the duration and severity of the clinical lytic symptoms (4). ACV is activated by the viral thymidine kinase and blocks viral DNA replication (4).

All herpesviruses replicate their genomes in the infected cell nuclei. In the nucleus, the HSV-1 genome can initiate either lytic replication or latent infection (5). The lytic infection is characterized by an ordered three steps of gene expression, immediate early (IE), early (E), and late (L) genes. This temporal cascade starts with the IE genes and then continues through E genes and DNA replication to the L gene expression (6). The tegument protein, viral protein 16 (VP16), activates the transcription of the IE genes following the entry of viral genomes to the nucleus. VP16 interacts with host factors to form a complex that allows transcription of the IE genes (7). Most IE genes play an important role in regulation of viral gene expression (5, 8). The E genes are mostly responsible for viral nucleic acid metabolism and viral replication. The onset of viral replication allows the expression of the L genes, which are mainly structural components that assemble the virions (9).

HSV-1 latency is established within the neuronal cell nuclei, where it is stably retained and is characterized by repression of lytic genes (2, 10–12). The viral locus encoding the latency-associated transcripts (LATs) contributes to repression of the lytic gene expression and can be detected during latency (13). During latency, viral DNA genomes become circular molecules (episomes) and bind host histones. When latent infection is established, the lytic genes are associated with chromatin, which uses histone modifications that are indicative of heterochromatin, and the lytic genes are silenced (14–17).

Spontaneous *in vivo* latency takes place almost solely in the native host’s peripheral sensory neurons or neurons of the autonomic sympathetic ganglia (3). Recently, we have shown *in vitro* that a small minority of nonneuronal cells can spontaneously maintain viral genomes in a latency-like state (18). The absence of good, spontaneous models for latency and reactivation led to many models that study latency by restricting viral lytic infection. Several small animal models that can recapitulate the complexity of whole organisms during latency were developed (11). *In vitro* models are useful for studying latency at the cellular level and identifying specific viral host interactions. *In vitro* models include latency or quiescence establishment in human or animal neuronal cells and nonneuronal human cells (Table 1). Other quiescence models use mutations in the viral immediate early genes (19, 20).

**TABLE 1:** Models of latency and quiescent infection of HSV-1 *in vitro*^*a*^

Model type	Cells	Latent-state induction	Reactivation method	Reference
Neuronal	SCG neurons	Acyclovir	Depletion of NGF using an anti-NGF antibody	50
	SCG neurons	Acyclovir	Inhibition of PI3K signaling (using LY294002)	21
	SCG neurons	NGF	NGF deprivation	51
	SCG neurons	NGF, acyclovir	NGF deprivation	52
	SCG neurons	Acyclovir, IFN	LY294002	22
	SCG neurons	Acyclovir	NGF deprivation	53
	SCG neurons	Acyclovir	Depletion of NGF using an anti-NGF antibody	54
Differentiated	hESC-derived neurons	Acyclovir	Growth factor withdrawal, PI3K inhibition, 34°C	55
	NIH-approved embryonic stem cell line	Low inoculum, acyclovir, and high-dose IFN-α	Sodium butyrate, a histone deacetylase inhibitor	56
	LUHMES	Acyclovir	PI3K inhibitor	57
Nonneuronal	HFFs	ara-C and elevated temperatures	Spontaneous reactivation after 5–11 days	58
	HFL-F cells	BVDU, acyclovir, IFN-α, 40.5°C	HCMV, 37°C	29
	Human embryonic lung cells	Cycloheximide for 24 h at 37°C, then 40.5°C	HCMV, 37°C	30
	Human diploid fibroblasts, human fetal lung cells	42°C	Superinfection of monolayers with viruses that express the HSV-1 ICP0	59
	Normal human diploid fibroblasts	Serum starved, heat shock, 41°C	Adenoviral vector	60
	HeLa/HB2	Spontaneous	Spontaneous	18

a A list of latency and quiescent models *in vitro* divided by the origin of cells being used is shown. The induction treatment and reactivation trigger are noted. SCG, superior cervical ganglion; NGF, nerve growth factor; PI3K, phosphoinositide 3-kinase; hESC, human embryonic stem cell; IFN-α, interferon alpha; LUHMES, Lund human mesencephalic; ara-C, cytosine arabinoside; HLF-F, human fetus lung fibroblast; BVDU, brivudine.

When a host undergoes stress such as fever or trauma, reactivation is usually triggered (3). Reactivation is a process where the latent HSV-1 genome reenters a lytic process and replicates to produce viral progeny. Reactivation has two phases. Phase I is reversible and may not continue to full productive reactivation, and most of the viral genes are expressed regardless of their lytic kinetics (21, 22). Phase II results in full reactivation that starts with the synthesis and activity of VP16, the viral transactivator, and the formation of productive progeny viruses and DNA replication (21–23). It is assumed that reactivations (either symptomatic or asymptomatic) are a major source of viral spread among individuals (24).

The number of latent genomes in an individual neuron range between less than 10 and more than 1,000 per cell (25–27). Little is known about the number and type of genomes that reactivate. Here, we set up a quantitative model to study the preferences of reactivation. We identified that, in most cases, only a single genome is reactivating. We were not able to detect significant preference for the onset of quiescent condition on reactivation. We observed that during quiescence, genomes are less likely to recombine. Further, reactivation-deficient viruses can reactivate only when another coinfecting genome reactivates.

## RESULTS

### Developing a quiescence system for reactivation studies.

**FIG 1:**
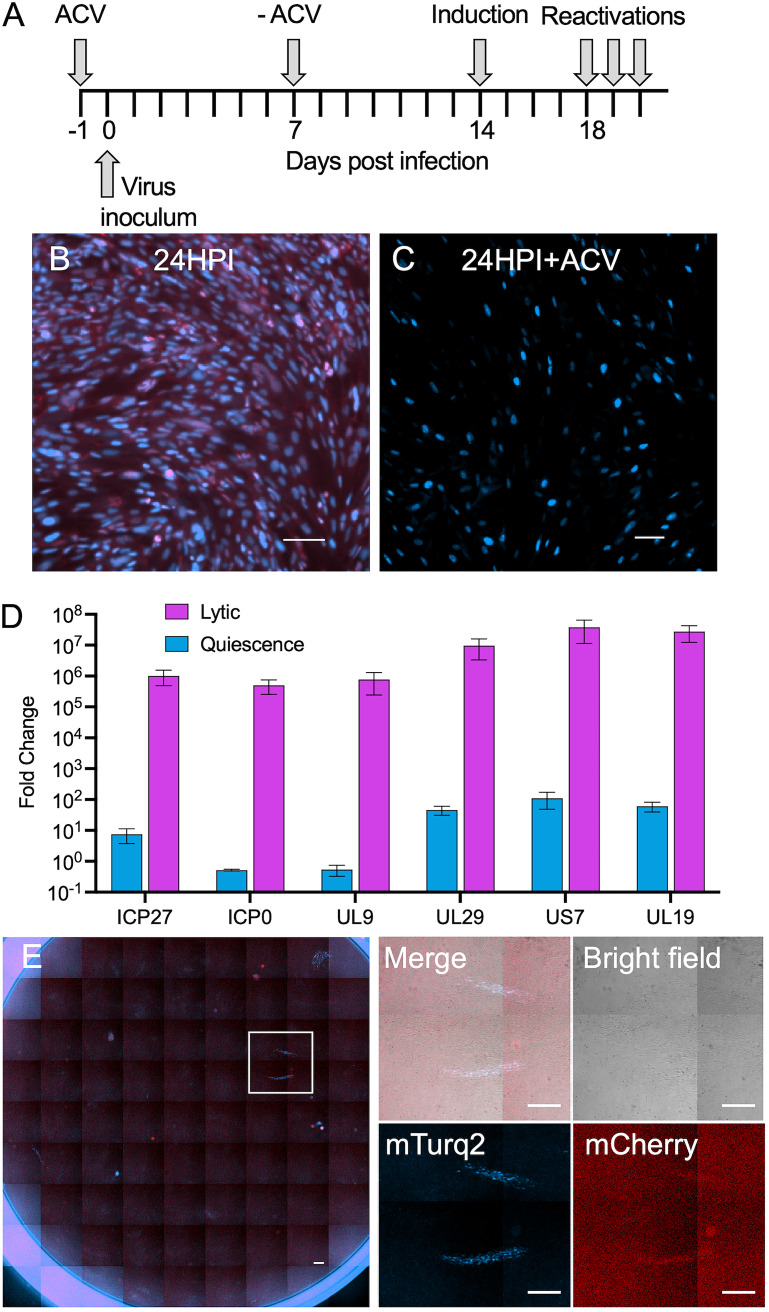
Establishment of quiescent infection system for reactivation using dual-colored virus. (A) Visual representation of timing of quiescent protocol. (B) Representative fluorescent image of lytic infection with HSV-1 OK41 at 24 hpi. Scale bar, 50 μM. (C) Representative fluorescent image of infection with HSV-1 OK41 in the presence of ACV at 24 hpi. Scale bar, 100 μM. (D) The fold change in viral immediate early (ICP0 and ICP27), early (UL9 and UL29), and late (UL19 and US7) gene expression between 18 hpi following lytic infection (purple) and quiescent state 3 days after removal of ACV (blue) was analyzed using qPCR. An average of two experiments each with two samples (each done in duplicates qPCR) is shown. Error bars represent SEM between the experiments; *n* = 4. (E) Representative fluorescent image of an entire well with 4 reactivation events of HSV-1 3 days posttreatment with HCMV. Scale bar, 500 μM. Magnification of the square area in panel D overlaid with a brightfield image is shown. Individual channels and a merged image are presented as indicated. Scale bars, 500 μM.

To study reactivations, we established a reproducible quiescence system (Fig. 1A). To calibrate the infection conditions, we infected human foreskin fibroblasts (HFFs) with a dual-color HSV-1 virus (OK41) that expresses mTurq2 (a cyan fluorescent protein [CFP]) under the immediate early cytomegalovirus (IE-CMV) promoter (expressed in similar kinetics as immediate early genes of HSV-1 [28] but independent of VP16 transactivation) and mCherry (a red fluorescent protein [RFP]) fused to the UL25 gene under the late native promoter. This allowed us to distinguish between acute infection, where both fluorophores are expressed, to quiescent infection in which only the CFP is expressed (Fig. 1B and C). Following infection of OK41 at a multiplicity of infection (MOI) of 2 in the presence of ACV, we were unable to detect late gene expression (i.e., red fluorescence), suggesting a quiescent state. Immediate early (ICP27 and ICP0), early (UL29 and UL9), and late (UL19 and US7) gene expression decreased by at least 5 log 3 days postremoval of the ACV compared to 18 hours postinfection (hpi) of lytic infection, suggesting very limited gene expression (if any) in the quiescent state (Fig. 1D).

To induce reactivation, the HFFs in the quiescent state were challenged by several methods. Some of the methods, including incubation at higher temperatures (39°C or 41°C) or with histone deacetylases inhibitor trichostatin A (TSA) treatment, did not result in significant reactivation above the control treatments. Next, we tested induction using human cytomegalovirus (HCMV) infection (as was previously done [29, 30]). Following HCMV infection at an MOI of 0.1, 3.3 reactivation events of the quiescent HSV-1 per well were detected by fluorescent viral plaques about 3 days post-HCMV infection (Fig. 1E). This is compared to mock-infected cells in which only 0.75 reactivation events per well were observed.

An alternative reactivation method was induced by expressing VP16 in the quiescently infected HFFs. The VP16 gene (see Materials and Methods) was delivered by a lentivirus infection. We observed 16.5 reactivation events per well following VP16 expression compared to 7.2 reactivation events per well following infection with the backbone lentivirus (not expressing VP16). Similar to the reactivations by HCMV infection, viral plaques were observed 3 to 4 days post-lentivirus transfection.

### A single viral genome reactivates from an individual cell.

**FIG 2:**
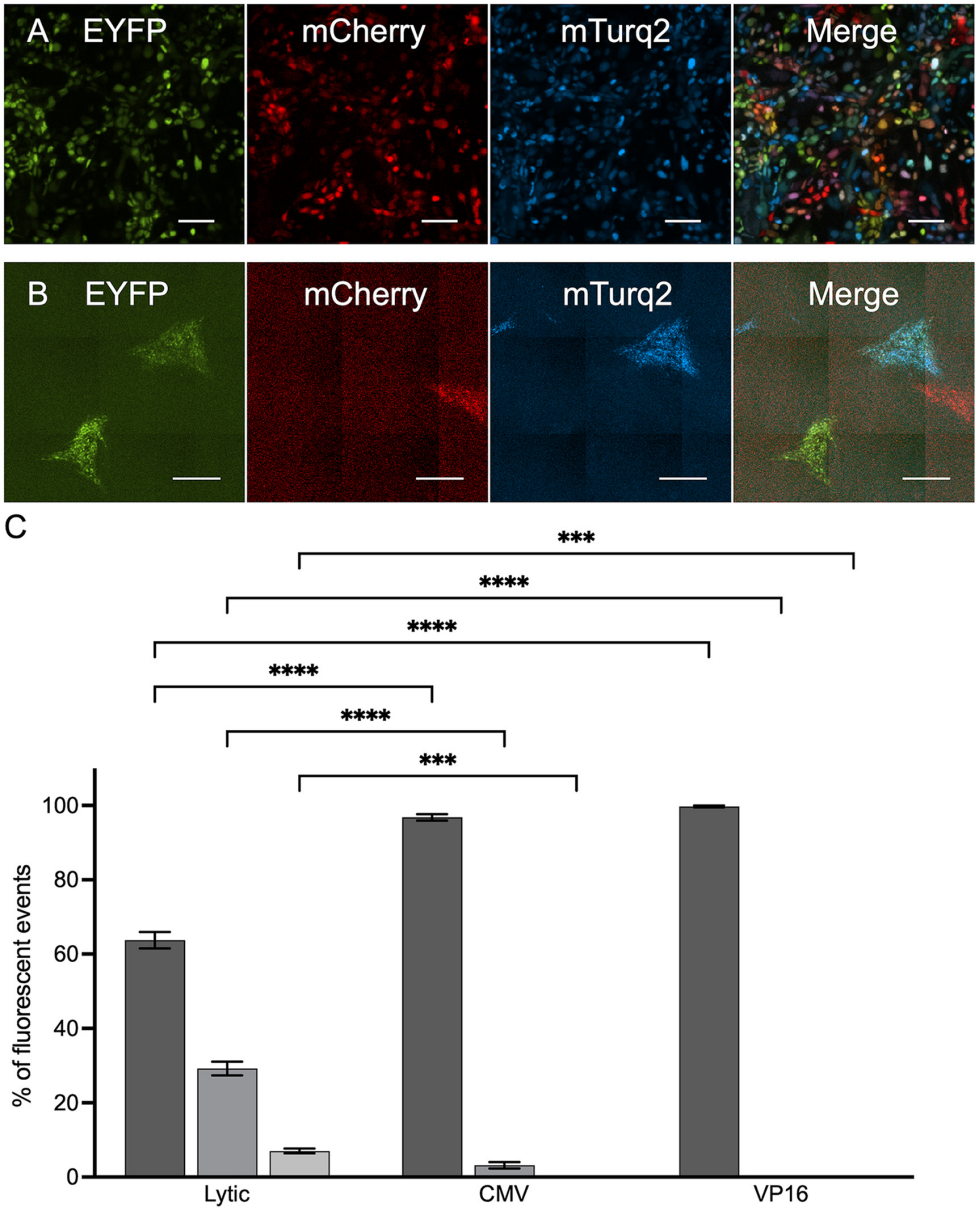
The number of viral genomes reactivating from an individual cell is very low. Immortalized HFF cells were infected at an MOI of 5 with a mixture of three HSV-1 recombinants, OK11, OK12, and OK22, each carrying a single fluorescent protein (mCherry, EYFP, and mTurq2, respectively). (A) A representative fluorescent image of 3-color lytic infection, individual fluorescent channels, and a merged image are presented as indicated. Scale bars, 100 μM. (B) Representative fluorescent image of quiescently infected HFF cells following reactivation 3 days posttreatment with HCMV. Four plaques (each originating from an individual reactivation) can be observed, one of each color and one that expresses both EYFP and mTurq2. Individual fluorescent channels and a merged image are presented as indicated. Scale bars, 500 μM. (C) For lytic infection, images from three experiments were used to calculate the number of cells expressing single, dual, or triple colors. At least 3,000 cells were analyzed from each experiment. For the reactivation assay, plaques after reactivation events by either HCMV or VP16 were analyzed to calculate the number of plaques expressing single, dual, or triple colors. Each bar represents three experimental repeats and is based on more than 200 plaques for each condition. Error bars represent SEM between the experiments; *n* = 3. ***, *P* < 0.001; ****, *P* < 0.0001.

We have previously shown that, on average, only a limited number of herpesvirus genomes are expressed and replicate in individual cells (28, 31). This was calculated from infection using a mix of three isogenic viruses, each carrying a different fluorescent protein, and measuring the amounts of single-, dual-, and triple-color infections (32). To identify the number of HSV-1 genomes that reactivate from a single cell, we combined the three-color infection assay and our model for quiescent infection in nonneuronal cells described above. HFF cells were coinfected with a mixture of three viral recombinants (OK11-red, OK12-yellow, and OK22-cyan) at an MOI of 5 in the presence or absence of ACV. In the absence of ACV, the cells were imaged 8 hpi, and the numbers of single-, dual-, and triple-color cells were counted (Fig. 2A and C). In the ACV-treated cells, the ACV was maintained for 8 days and removed for 3 days. Reactivation was induced, using either HCMV infection or VP16 transfection. In both reactivation methods, more than 95% of the reactivating plaques expressed only a single color, while some expressed two colors, and none of the reactivating plaques expressed all three colors (Fig. 2B and C). HCMV infection induced more coreactivations than VP16; however, the differences were not significant. In contrast, in the lytic infection, a statistically significant difference from the reactivation assay was observed, as 62.7% single-, 30.2% dual-, and 7.1% triple-colored cells were counted. The mathematical model we have developed considers the possibility of multiple expressions by one type of genome and provides the most likely average number of genomes that are expressed in a single cell/plaque (32). Using this model, we estimate that an average of 1.26 and 1.21 genomes initiated reactivation per cell for HCMV and VP16 inductions, respectively, compared to an average of 2.0 genomes initiating expression per cell during the lytic infection. These results indicate that reactivation initiates almost exclusively from a single genome per cell.

### Timing of infection does not influence the probability of reactivation among individual viral genomes within a cell.

**FIG 3:**
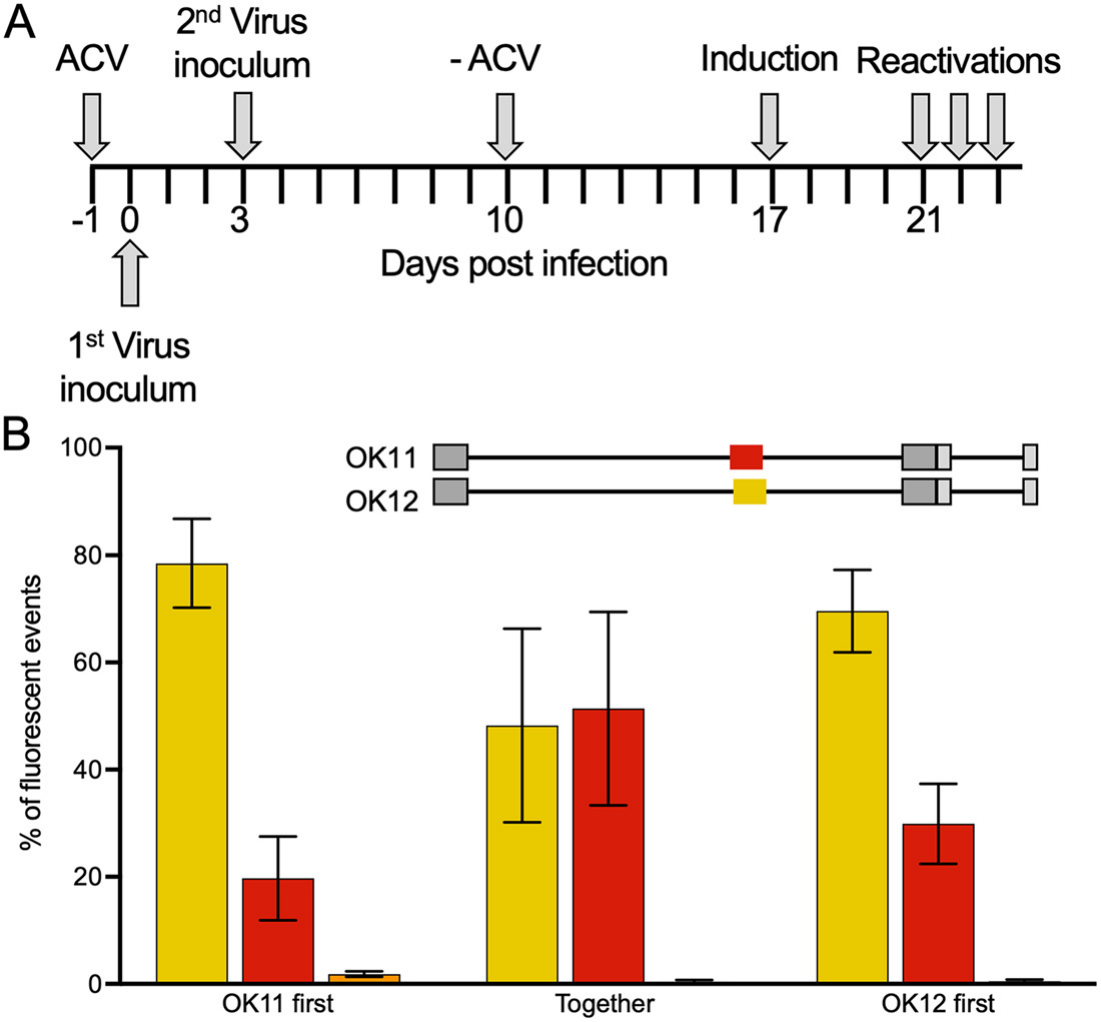
Entry time to latent infection does not affect viral ability to reactivate. (A) Visual representation of timing of sequential quiescent protocol. (B) HFF cells were quiescently infected with two fluorescent protein gene-expressing HSV-1 recombinants (schematic illustration of the color and place of the reporter genes, above the graph) at an MOI of 5 at the same time or 3 days apart with a second virus expressing different XFPs (infected at the same MOI). Emerging plaques following reactivation induced using VP16 were used to calculate the number of plaques expressing one or two colors. Bars are color coded like the plaques (yellow, red, or both in orange). Each bar represents four experiments and is based on more than 150 plaques for each condition. Error bars show SEM between the experiments; *n* = 4.

Following our finding that in most cases, only one genome reactivates from an individual cell, we question whether some genomes are more likely to reactivate than others. We speculated that genomes that enter quiescence earlier are less likely to reactivate, as they will be more repressed with time, as it was observed that shedding and reactivation are reduced with time (33). To test the effect of timing on viral ability to reactivate, we coinfected HFF cells with two viral recombinants (OK11-red, OK12-yellow) either at the same time or 3 days in between in the presence of ACV. To ensure that our results are not biased by differences among the recombinants, infection was initiated with either OK11 or OK12 first. The infected cells were incubated 7 days after the second infection in the presence of ACV; then, the ACV was removed, and the cells were maintained in acyclovir-free medium for 3 more days. We reactivated the quiescent cells by using transfection of the VP16-expressing lentivirus and measured the ratio of reactivating viruses in each color (Fig. 3A).

We compared the ratio of progeny viruses from infection with either of the viruses earlier to cells that were coinfected together (Fig. 3B). As we have seen with the three-color infection, reactivation of more than one virus was extremely rare (less than 1% in each condition). The rates of yellow and red reactivations differ within the different conditions among experiments (large error bars) and among the different conditions (Fig. 3B). However, no significant differences were detected between cells infected according to the altering timing protocol and cells infected with both recombinants simultaneously. In our experimental system, we were unable to show that the probability of reactivation for an individual viral genome depends on the time the virus enters the quiescent state.

### Limited recombination events during quiescence and reactivation.

Recombination among herpesviruses is very common and is considered a major driving force of viral evolution (34). We and others have previously shown that fluorescent protein (XFP)-expressing HSV recombinants are a useful tool to measure recombination rates (35, 36). We set up experiments to test whether recombination could occur during the quiescent state or reactivation.

**FIG 4:**
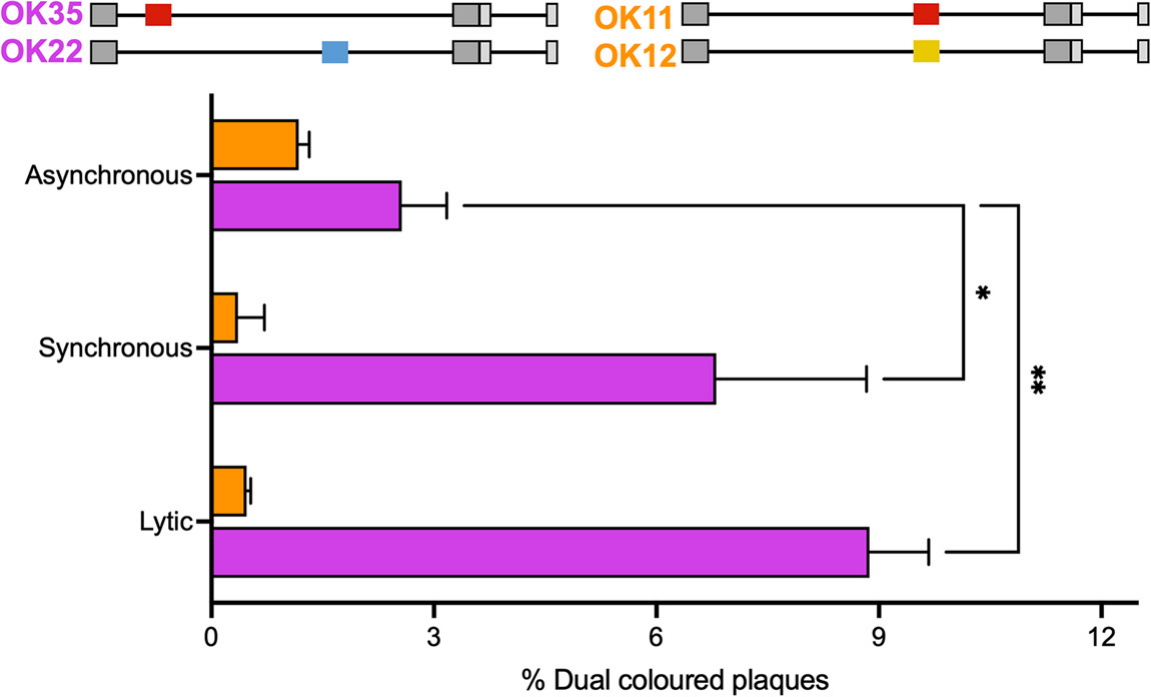
Recombination rates during latent infection. HFF cells were lyticly or quiescently infected with either of two sets of XFP-expressing HSV-1 recombinants (as illustrated above the graph) at an MOI of 5, at the same time (lytic and synchronous) or 3 days apart (asynchronous). The OK35 and OK22 set (purple) has fluorescent markers in different sites of the genome, whereas the OK11 and OK12 set (orange) has fluorescent markers in the same sites of the genome. Emerging plaques following reactivation induced using VP16 were used to calculate the number of plaques expressing one or two colors. The percentage of dual-color plaques out of the total plaques observed is shown. Each bar represents three experiments and is based on more than 300 plaques for each condition. Error bars represent SEM between the experiments; *n* = 3 for OK35 and OK22, and *n* = 4 for OK11 and OK12. *, *P* < 0.05; **, *P* < 0.01.

To measure recombination rates following the quiescent state, we coinfected cells with two viral recombinants, each expressing a different XFP at a different site of the genome (OK22 and OK35). We analyzed recombination rates among reactivation events by measuring the amount of dual-colored plaques out of the total number of fluorescent plaques (Fig. 4). To distinguish if recombination is due to processes taking place prior to the establishment of quiescence or during reactivation, we repeated the experiments of the timing conditions to detect differences in recombination rates once the viruses did not enter at the same time (asynchronous infection). We compared the rate of recombination to lytic coinfection under the same conditions. Our results show that asynchronous latent-like infection resulted in lower dual-colored plaque rates after reactivation (3.0% of all plaques) than those observed following synchronous latent-like infection (6.8%) or after the lytic infection (8.9%). To ensure that dual-colored plaques are the results of recombination events, we compared our results to the percentage of dual-colored plaques following coinfection in identical conditions with viral recombinant, each expressing a different XFP at the same site of the genome (OK11 and OK12). The percentage of dual-colored plaques following coinfection with these recombinants did not pass 1.2%, indicating that most of the dual plaques observed following the recombinants with fluorescent genes at different sites are likely due to recombination. These results suggest that intergenomic recombination events are less frequent after the quiescent state was established and during reactivation than recombination rates following lytic infection.

### The reactivation-deficient mutant requires coinfection.

Infected cell protein 0 (ICP0) is an immediate early protein of HSV-1 with ubiquitin ligase activity. ICP0 is required for efficient lytic infection and reactivation from latency (37). To test complementation among quiescent viral genomes, we used a viral recombinant (OK29) that carries a known deletion in ICP0 and carries the red fluorescent protein (31). When infected alone, OK29 did not reactivate at all.

**FIG 5:**
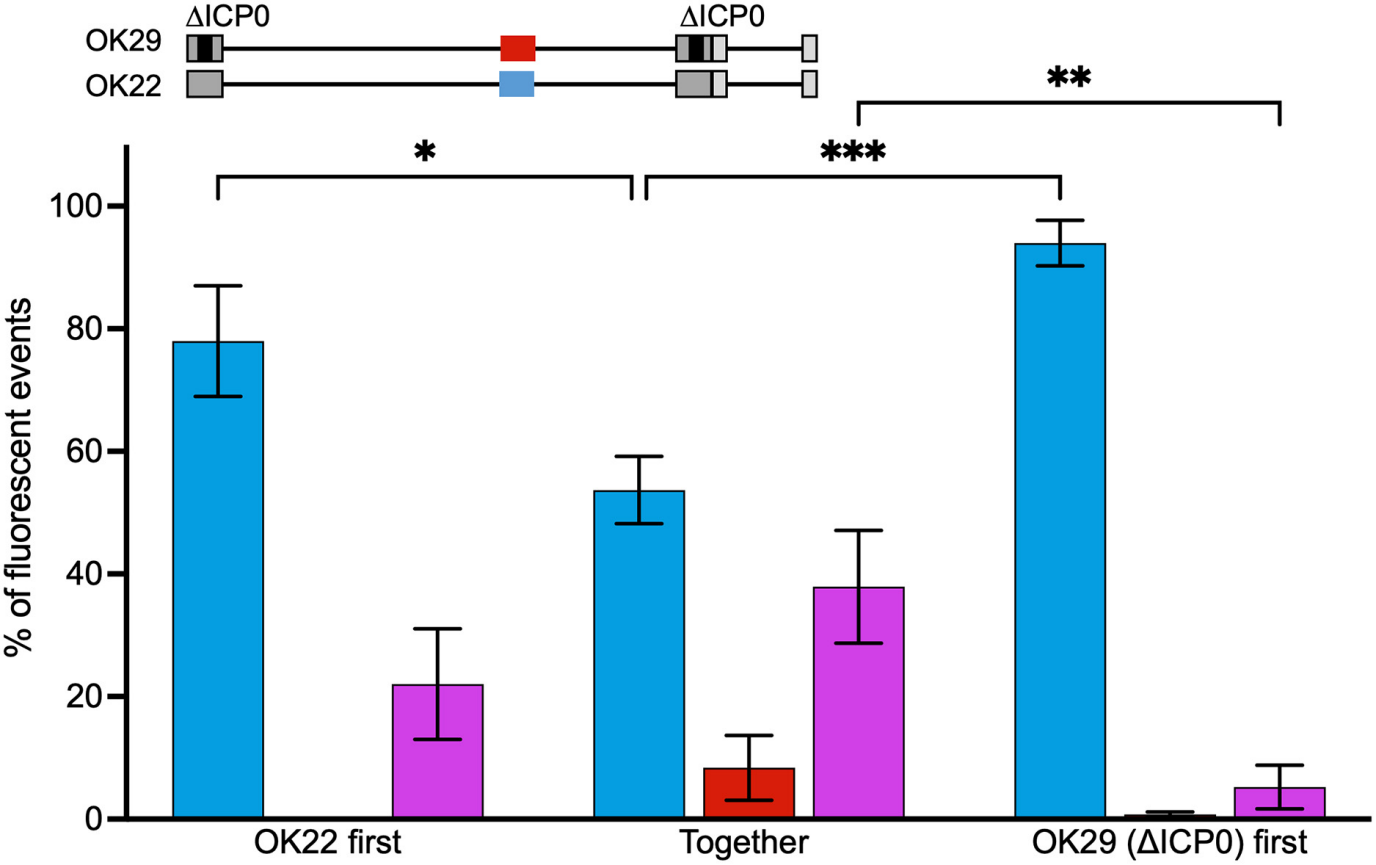
Reactivation of viral mutant requires the presence of complementing viral genomes. HFF cells were quiescently infected with an mCherry-expressing HSV-1 mutant (OK29-ICP0 deletion), at the same time or 3 days apart with a second complementing virus (OK22-WT) expressing different levels of mTurq (as illustrated above the graph) at an MOI of 5. Emerging plaques following reactivation induced using VP16 were used to calculate the number of plaques expressing one or two colors. Bars are color coded like the plaques (blue, red, or both in purple). Each bar represents three experiments and is based on more than 50 plaques for each condition. Error bars represent SEM between the experiments; *n* = 3. *, *P* < 0.05; **, *P* < 0.01; ***, *P* < 0.001.

We coinfected cells with the OK29 recombinant and a wild-type recombinant carrying the cyan fluorescent protein (OK22). After the establishment of quiescence of both viruses, either synchronously or with 3 days difference, the ratio of progeny viruses in each color was measured following induction of reactivation. Our results show that when infected with a wild-type recombinant, reactivating plaques appeared; most plaques contain only the wild-type recombinant (50% to 90% of plaques; Fig. 5). When infection was done simultaneously, we observed 8.3% of plaques carrying the red fluorescent protein only. We speculate that these reactivations are probably the wild-type genome that recombined with the red fluorescent protein, as it is seen almost exclusively in the simultaneous infection, a condition that favors recombination during quiescent state (Fig. 4). In contrast to the coinfection with two wild-type strains (Fig. 2 and 3), dual-color plaques were observed frequently (37.9% for synchronous infection, 22.0% for the wild type first, and 5.2% for the mutant first). These results suggest that either entry to the quiescent state or reactivation of mutant viruses can be supported by competent virus in the same cell.

## DISCUSSION

The reactivation of herpesviruses is a major source of their successful strategy as pathogens. Here, we set up an experimental nonneuronal quiescent infection system to obtain information on the likelihood of reactivation of coinfecting viral genomes. We used fluorescent-expressing viruses to identify that, in most cases, only a single viral genome is reactivating. The high probability of a single genome reactivating raised the question of whether there is any preference for the genome being reactivated. Our results indicate that the timing of entry to quiescence does not affect the probability of reactivating. On the other hand, reactivation events of mutant viral genomes are less likely and require coreactivation with a complementing genome. Taken together, we propose that sporadic reactivation events occur stochastically at the single-genome level.

Many different types of HSV-1 models of latency or quiescent infection were explored previously. Most of the *in vitro* models (Table 1) are based on inhibiting the viral replication (using ACV as we have done here) and allowing the epigenetic silencing of viral gene expression. The use of a chain-termination drug to induce the quiescent state, while commonly used, may affect the state of the quiescent genomes, as they may not be fully episomal as suggested for natural latent genomes (38). Further, it is likely that some of the entering genomes, which were suppressed before replication could initiate (5, 39), may be repressed by common mechanisms compared to genomes that initiated replication and were stopped. The stalled replicating is likely to induce a stronger DNA damage response, and therefore, these genomes that initiated replication might be less likely to reactivate.

All models of latency or quiescent infection fail to recapitulate the entire complexity of natural *in vivo* latency, but they are beneficial for studying specific properties of latency and reactivation (11). Many of these studies focus on cells of neuronal origin, as they are the only cells that were identified as sites of HSV-1 latency *in vivo*. However, our recent findings that in nonneuronal culture cells, a latency-like state can be reached spontaneously suggest that some of the mechanisms involved in latency establishment (including chromatinization of the latent genomes) and reactivations are conserved in nonneuronal cells (18). We use a quiescent model based on human fibroblasts that, despite its limitations, allows the detection of hundreds of reactivation events to quantify the preferences of the reactivation process.

We measured reactivation by counting the formation of plaques around the reactivating cells. This was possible due to the low rate of reactivation per well we observed, thus decreasing the possibility of adjacent reactivations being counted as one plaque. The very limited number of plaques with dual colors strengthens the notion that each plaque represents only one reactivation process. Counting the plaques ensured we count only reactivations that led to infectious progeny viruses. We have tested two methods of reactivation, either infection with HCMV, a betaherpesvirus with a relatively slow replication cycle, or transfection of a lentivirus expressing VP16, the HSV-1 transcription activator of the immediate early genes. The induction of HSV-1 reactivation by HCMV was shown in the 1980s; however, the mechanism was not established (29, 30). It was shown that HCMV infection induces the disruption of promyelocytic leukemia (PML) nuclear bodies (40) and PML bodies are involved in controlling HSV-1 latency (41), suggesting a possible factor contributing to the induction of reactivation by HCMV. VP16 is suggested to induce phase II of the reactivation process. In our system of quiescent infection, reactivation with VP16 occurred more often and induced more one-color reactivation events than HCMV reactivation (Fig. 1 and 2). We therefore continued with the VP16 induction throughout the study.

Using the three-color infection model, we have found that only a limited number of incoming herpesvirus genomes initiate expression and replication during lytic infection in a given cell (31, 32, 42). Our results were further validated using a genetic barcoding system (28). Here, we utilized the system to estimate the number of genomes being reactivated. We assume that under our current conditions, ~40% of cells have expressed more than one genome, representing the potential percentage of quiescent cells carrying more than one genome. We have found that the number of genomes reactivating is significantly lower than during acute infection (Fig. 2). For the gamma herpesviruses, multiple copies of genomes can be found in latently infected cells both *in vivo* and *in vitro* (43, 44); thus, it will be interesting to determine if such a phenomenon occurs in these viruses’ reactivations.

It was suggested that genetically diverse HSV-1 strains (KOS63 and KOS79) did reactivate in a single person at different times (45), raising the possibility that reactivation might select a different viral genome each time. We set up dual-color quiescent infection to determine if there is any preference for genomes reactivating. We tested if genomes entering later to the dormant state are preferably reactivating. Our results (Fig. 3) suggest that, in our settings, timing did not significantly influence the probability of reactivation. The nonconsistent differences among the two coinfecting viruses’ reactivations observed within single experiments further support a random process without preference. In our experimental setting, the difference between the infections was 3 days, and it is possible that much longer time differences (that are hard to reach with *in vitro* settings) are required for forming a strong preference.

Recombination was found in many HSV-1 isolates (46), although it is known that HSV-1 has several mechanisms for superinfection exclusion (47). One can predict that our method of inducing quiescence by the DNA chain termination by acyclovir may increase recombination once replication is reinitiated. On the other hand, HSV-1 genomes are not known to replicate during latency, and replication is coupled with recombination (48); thus, during the quiescent period, recombination events should be limited. Our results raise the possibility that asynchronous infection to the quiescent state leads to a lower likelihood of recombination than synchronous coinfection (Fig. 4), suggesting that most recombination events occur during the establishment of the quiescent condition and not during the dormant period or the reactivation. The reduced rates of recombination events during reactivation corroborate our finding that, almost exclusively, only one viral genome reactivates per cell (Fig. 2).

The HSV-1 ubiquitin ligase ICP0 is required for efficient lytic infection and reactivation by degrading or modulating the functions of host proteins involved in antiviral defenses (37). ICP0 is likely to work in *trans*, as it directly affects host proteins in the nucleus and cytoplasm. Our results that HSV-1 deletion of ICP0 can reactivate only following coinfection with ICP0-positive virus further corroborate this idea (Fig. 5). Our results do not distinguish if the mutant requirement is during the establishment of the quiescent state or during the reactivation *per se*. However, under our experimental assay, these are the only conditions demonstrating significant amounts of coreactivations. Why coreactivations occur only when a viral mutant is unable to reactivate by itself? We speculate that once the viral genome reactivates, it takes over the cell; however, once a mutant genome reactivates and is unable to complete the reactivation process, it provides the opportunity for other genomes in the cell to both reactivate and reactivate together.

Taken together, our findings support that reactivation events are random and rare. We have recently suggested, for lytic infection, the single-genome hypothesis in which each viral genome that enters the cell can have a different fate than the other genomes around it (5). To enter a latent state, all entering genomes must become quiescent, whereas even a single genome that is able to initiate lytic infection will determine the fate of the cell to lytic infection. Here, we observe a similar phenomenon during reactivation that it is enough that only one genome will reactivate to start the process. Our results agree with the low likelihood of reactivation observed *in vivo*. We hypothesize that all genomes are maintained in a quiescent state and are actively resuppressed even during phase I of reactivation; therefore, the probability of each genome undergoing complete reactivation is low. The notion that latency is noisier than originally predicted but reactivations are still rare further support this model (49). By improving our technical abilities to identify changes at the single-genome level within the latent cell, a clearer view of the events required for reactivation will emerge.

## MATERIALS AND METHODS

### Cells.

All experiments were performed using human immortalized foreskin fibroblasts (HFFs) that were immortalized by hTERT transfection (kindly provided from the Sara Selig lab). All viruses were grown and tittered on African green monkey kidney epithelial cells (Vero cells; ATCC CCL-81) or, in the case of ICP0 mutant viruses, human female osteosarcoma cells (U2OS cells; ATCC HTB-96). 293FT cells, a variant of the human kidney cell line 293, expressing simian virus 40 (SV40) large T antigen (provided by the Chen Luxenburg lab), were used for obtaining the VP16-expressing lentivirus. All cells were grown with Dulbecco’s modified Eagle medium (DMEM ×1; Gibco), supplemented with 10% fetal bovine serum (FBS; Gibco) and 1% penicillin and streptomycin (10,000 units/mL and 10 mg/mL, respectively; Biological Industries, Israel).

### Viruses.

All viruses are derivatives of HSV-1 strain 17+. Viral recombinants OK11, OK12, and OK22 carry a single fluorescent protein (mCherry, enhanced yellow fluorescent protein [EYFP], and mTurq2, respectively) with a nuclear localization tag under the CMV promoter between UL37 and UL38 genes (42, 47). OK39 was constructed to carry mCherry gene fused in-frame within the UL25 gene after the 50th amino acid of the viral protein. OK41 was constructed using dual infection and recombination between OK22 and OK39. Viral recombinant OK29 is an ICP0-null mutant with pOK11 mCherry-nuclear localization signal (NLS) from the CMV promoter between the UL37 and UL38 genes (31). The viral recombinant OK35 carries the mCherry gene under the CMV promoter between the UL3 and UL4 genes,(36). CMV AD169 was kindly provided by Noam Stern-Ginossar.

### VP16 modified protein.

**FIG 6:**
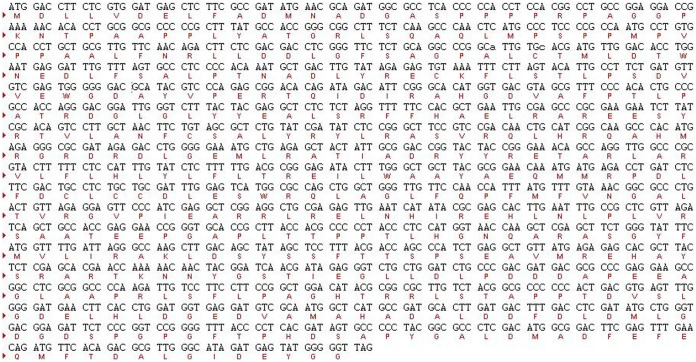
Modified VP16 gene. The DNA and amino acid sequence of the modified VP16 gene used for reactivation are presented.

The viral protein VP16 was modified in a way that the sequence is different from the original viral sequence (to reduce the possibility of recombination between the plasmid and the viral genome, required for a different project in the lab), but the protein formed is identical to the viral one (Fig. 6). The sequence was synthesized as gBlocks (IDT) and inserted into a pcDCMV-EF106 plasmid.

### Lentivirus assembly.

293FT cells were seeded in a 6-well plate (2 × 10^6^ cells per well) 24 h prior to transfection. One hour prior to transfection, the medium was changed to 1 mL DMEM ×1 (Gibco), supplemented with 1% penicillin and streptomycin (Biological Industries Israel). Three 3rd-generation lentiviral plasmids and the recombinant VP16 modified plasmids were mixed (VP16 modified, 0.4 μg; pLP1, 0.26 μg; pLP2, 0.1 μg; pVSV-G, 0.14 μg) in DMEM with PolyJet (Signagen Laboratories; catalog number SL100688) reagent according to the manufacturer’s recommendation and added to the wells. After incubation for 24 h at 37°C, the medium was discarded and replaced. After incubation for 48 and 72 h posttransfection, the supernatant containing lentiviruses was collected and kept at 80°C.

### Microscopy.

To estimate the number of HSV-1 genomes reactivating from individual cells, we obtained images using a Nikon Eclipse Ti-E epifluorescence inverted microscope (Nikon, Tokyo, Japan). Each experimental condition (different viruses, timing of infection, lytic or latent infections) was replicated in at least 3 wells, and each experiment was performed at least twice.

### The quiescent state model.

HFF cells were treated with ACV (Sigma-Aldrich; catalog number PHR1254-1G) for 24 h before infection with different viruses (according to the experimental conditions required) carrying different fluorescent proteins, including OK41 (mCherry and mTurq2) OK11 (mCherry), OK12 (EYFP), OK22 (mTurq2), OK29 (mCherry), and OK35 (mCherry). The infected cells were maintained in specific conditions, 37°C with ACV-containing medium for 7 days. Next, the ACV was removed from the medium, and the quiescently infected cells were maintained in regular medium for 5 to 7 days until reactivation was induced. Reactivation was induced by infecting those cells with HCMV or lentivirus expressing VP16 transfection. Three to 5 days postreactivation, the plates were scanned using Nikon Eclipse Ti-E epifluorescence inverted microscope, and plaques were counted according to the fluorescent protein expressed.

### Reverse transcriptase quantitative PCR (RT-qPCR).

To assess HSV-1 gene expression during quiescence using qPCR, RNA was first isolated from cells. HFF cells were seeded on 12-well plates and, 24 h later, infected with OK41 at an MOI of 5. Lytic samples were taken 18 h postinfection. The quiescence samples were incubated for 7 days postinfection in the presence of ACV and for an additional 3 days without ACV. At the end of the incubation period, the medium was removed, and TRIzol (BioTri; Bio-Lab) was added and incubated at room temperature for 15 min. The sample was then collected and stored at −80°C until RNA purification. RNA was isolated using Direct-zol RNA miniprep kit (Zymo) according to the manufacturer’s protocol, including a DNase I step. Purified RNA was then reverse transcribed to cDNA using Quantabio cDNA kit according to the manufacturer’s protocol. qPCR was performed (CFX, Bio-Rad) using 2× Sybr green master mix (Applied Biosystems). Each cDNA sample was analyzed for 2 viral immediate early (ICP0 and ICP27), early (UL9 and UL29), and late (UL19 and US7) genes and a cellular housekeeping gene (GAPDH [glyceraldehyde-3-phosphate dehydrogenase]). The sequences for primers (from IDT) for viral genes and the cellular housekeeping gene appear in Table 2. Fold change was calculated by normalizing each sample to the GAPDH levels and then comparing lytic and quiescence sample results to uninfected cells.

**TABLE 2:** List of primers used in this study for viral gene expression

Primer	Sequence (5′→3′)
ICP0 fwd	TCTTCCTCCTCTGCCTCTTC
ICP0 rev	AGGGAGGTTTCCTCTTGTCT
ICP27 (UL54) fwd	CCTTTCTCCAGTGCTACCTG
ICP27 (UL54) rev	GCCAGAATGACAAACACGAAG
UL9 fwd	TTTGTGACCTGGGAGAACTG
UL9 rev	ACTGATTTACATGGACGGCTC
UL29 fwd	GAAGGTGCATAGGTTACAGGG
UL29 rev	GCCAAGATGCTGTTTTACCTG
UL19 fwd	GACCGCTTTGTGACTGAGAA
UL19 rev	CTGGGTGAGCGTGAAGTTTA
US7 fwd	CACGGTCAGTCTGGTATCAA
US7 rev	CCCGAGAATAAGCAGGTCTT
GAPDH fwd	ACCCACTCCTCCACCTTTGA
GAPDH rev	CTGTTGCTGTAGCCAAATTCGT

### Statistical analyses.

All statistical analyses were performed using GraphPad Prism 9 software. All fluorescence experiments are presented as means ± standard errors of the mean (SEM). All data were analyzed by two-way analysis of variance (ANOVA), followed by Tukey’s multiple-comparison test. A *P* value of <0.05 was considered statistically significant.
